# 3D virtual histology of human pancreatic tissue by multiscale phase-contrast X-ray tomography

**DOI:** 10.1107/S1600577520011327

**Published:** 2020-10-23

**Authors:** Jasper Frohn, Diana Pinkert-Leetsch, Jeannine Missbach-Güntner, Marius Reichardt, Markus Osterhoff, Frauke Alves, Tim Salditt

**Affiliations:** a Institute for X-ray Physics, Universität Göttingen, Friedrich-Hund-Platz 1, 37077 Göttingen, Germany; b Institute of Diagnostic and Interventional Radiology, University Medical Center Göttingen, Robert Koch Strasse 40, 37075 Göttingen, Germany; c Max Planck Institute for Experimental Medicine, Group of Translational Molecular Imaging, Hermann-Rein-Strasse 3, 37075 Göttingen, Germany; d Clinic of Hematology and Medical Oncology, University Medical Center Göttingen, Robert Koch Strase 40, 37075 Göttingen, Germany; e Multiscale Bioimaging: From Molecular Machines to Networks of Excitable Cells (MBExC), University Medical Center Göttingen, Robert Koch Strase 40, 37075 Göttingen, Germany

**Keywords:** X-ray tomography, phase retrieval, 3D histology, biopsy, pancreas

## Abstract

A multiscale three-dimensional (3D) virtual histology approach is presented, based on two configurations of propagation phase-contrast X-ray tomography, which have been implemented in close proximity at the GINIX endstation at the beamline P10/PETRA III (DESY, Hamburg, Germany). This enables the 3D reconstruction of characteristic morphological features of human pancreatic normal and tumor tissue, as obtained from cancer surgery, first in the form of a large-scale overview by parallel-beam illumination, followed by a zoom into a region-of-interest based on zoom tomography using a Kirkpatrick–Baez mirror with additional waveguide optics. To this end 1 mm punch biopsies of the tissue were taken. In the parallel tomography, a volumetric throughput on the order of 0.01 mm^3^ s^−1^ was achieved, while maintaining the ability to segment isolated cells. With a continuous rotation during the scan, a total acquisition time of less than 2 min was required for a full tomographic scan. Using the combination of both setups, islets of Langerhans, a three-dimensional cluster of cells in the endocrine part of the pancreas, could be located. Cells in such an islet were segmented and visualized in 3D. Further, morphological alterations of tumorous tissue of the pancreas were characterized. To this end, the anisotropy parameter Ω, based on intensity gradients, was used in order to quantify the presence of collagen fibers within the entire biopsy specimen. This proof-of-concept experiment of the multiscale approach on human pancreatic tissue paves the way for future 3D virtual pathology.

## Abstract 2

This paper presents propagation-based phase-contrast tomography in two configurations at the beamline endstation GINIX, demonstrated on the application of 1 mm human pancreatic tumor tissue biopsies.

## 1. Introduction   

The potential of propagation-based X-ray phase-contrast tomography for three-dimensional (3D) virtual histology has been pointed out by a number of recent studies (Saccomano *et al.*, 2018 ▸; Töpperwien *et al.*, 2018 ▸; Khimchenko *et al.*, 2016 ▸; Albers *et al.*, 2018 ▸; Dejea *et al.*, 2019 ▸; Ding *et al.*, 2019 ▸; De Clercq *et al.*, 2019 ▸; Strotton *et al.*, 2018 ▸; Mei *et al.*, 2020 ▸). Notably, 3D virtual histology by phase-contrast X-ray tomography is able to extend classical histological two-dimensional (2D) approaches, which are based on thin sections and light microscopy, into a full 3D visualization with isotropic resolution and without destructive slicing of the specimen. The interaction of X-rays with the object is described by the continuous complex-valued index of reflection 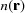 = 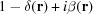. The particular advantage of phase contrast for low-absorbing materials such as biological tissue derives from the fact that for X-ray energies of 10 keV to 100 keV the absorptive component β is orders of magnitude smaller than the refractive component δ (Nugent, 2010 ▸). Contrast is formed by transformation of the phase shifts into measurable intensity variations by self-interference of the exit wave during free-space propagation between sample and detector (Paganin & Nugent, 1998 ▸; Cloetens *et al.*, 1999*a* ▸). By careful optimization of photon energy, illumination function, and phase-retrieval algorithms, the phase sensitivity suffices to probe also the intrinsic electron density variations of unstained tissue, for example tissue embedded in paraffin, ethanol, or even aqueous buffer (Töpperwien *et al.*, 2019 ▸). Compared with other full-field phase-contrast techniques, *e.g.* based on conventional grating interferometry or analyzer crystals, propagation-based imaging (PBI) can reach a resolution below optical microscopy (Khimchenko *et al.*, 2018 ▸).

Apart from non-destructiveness, isotropic resolution, quantitative and label-free contrast mechanisms, PBI has also a particular potential for achieving high volume throughput and for visualizing histological structure on multiple length scales. However, to bring this to reality, particular instrumentation and optical settings are required, as we explore and demonstrate in this work. Generally, synchrotron endstations are used for PBI in only one of two different configurations: (i) parallel-beam tomography, covering a large field of view (FOV), with resolution limited by the detector pixel size, *e.g.* the endstation ID19 of the European Synchrotron Radiation Facility (ESRF) (Weitkamp *et al.*, 2010 ▸) or the endstation TOMCAT of the Swiss Light Source (SLS) (Stampanoni *et al.*, 2007 ▸; Marone *et al.*, 2011 ▸), and (ii) cone-beam geometry, acquiring high-resolution tomograms with voxel sizes in the range 20 nm to 300 nm (Cloetens *et al.*, 1999*a* ▸; Mokso *et al.*, 2007 ▸; Bartels *et al.*, 2015 ▸), based on the geometrical magnification of the pixel size. This regime is typically characterized by small Fresnel numbers *F*, in the so-called holographic regime, where phase sensitivity is particularly high but phase retrieval can sometimes become a challenge (Lohse *et al.*, 2020 ▸). Beamline endstations running in this configuration are for example ID16a at ESRF (Da Silva *et al.*, 2017 ▸) or the GINIX endstation at PETRA III. A voxel size below 50 nm can easily be achieved by adjustment of the defocus distance (Bartels *et al.*, 2015 ▸; Krenkel *et al.*, 2017 ▸; Töpperwien *et al.*, 2017*a* ▸). Although both configurations had been run in a multiscale scheme [parallel beam (Dejea *et al.*, 2019 ▸) and cone beam (Bartels *et al.*, 2015 ▸)], to our knowledge this is the first time that both configurations have been combined for a multiscale approach.

The goal of the present work is threefold: First, we want to demonstrate how PBI can be carried out on the same instrument in parallel- and cone-beam setting, in such a way that different scales of the same sample can be investigated in close temporal and spatial proximity during the same beam time. Second, we want to demonstrate high volumetric flow rate (volume throughput) on the order of 0.01 mm^3^ s^−1^ with sub-micrometre pixel size and maintaining the ability to segment single cells. Third, we want to apply 3D virtual histology to human biopsies taken from surgery with the goal of extending conventional histopathology to full 3D and quantitative image analyis.

A full 3D imaging and automated digital pathology could provide new insights into patho-mechanisms and etiology of diseases. It may also serve for improved diagnosis, *e.g.* for subclassification of tumors thereby supporting clinical decisions. Even if future pathology applications might preferably be based on advanced laboratory micro-computed tomography (µ-CT) instrumentation for reasons of accessibility, one would probably still need synchrotron PBI for comparison and verification.

In this spirit, our group has previously shown that *post mortem* analysis of human tissue autopsies can be carried out at a liquid-anode µ-CT instrument, but required ground truth verification of segmentation parameters by synchrotron radiation (Töpperwien *et al.*, 2018 ▸).

As proof-of-concept for this multiscale histology approach, we choose tissue biopsies of human pancreatic cancer tissue (Kamisawa *et al.*, 2016 ▸). Pancreatic cancer is the fourth leading cause of cancer death and has the lowest five-year relative survival rate of all cancer types, for example (9%) in the USA (Siegel *et al.*, 2019 ▸). The most common pancreatic tumor, pancreatic ductal adenocarcinoma (PDAC), accounts for about 90% of the cases and is characterized by an invasive mucin-producing neoplasm of epthelial origin that causes an intense stromal desmoplastic reaction (Hruban, 2010 ▸). Morphologic criteria such as irregular arrangement of tubular glands, embedded in desmoplastic stroma, and signs of inflammation are characteristic features identified by conventional histology thereby helping to diagnose PDAC (Haeberle & Esposito, 2019 ▸). Since conventional histology requires slicing of the sample with several micrometre thickness, the 3D resolution perpendicular to the slice direction is limited by the slice thickness. 3D virtual histology can overcome this limitation and can hence probe the morphology more completely and at higher resolution (Bartels *et al.*, 2015 ▸; Khimchenko *et al.*, 2016 ▸, 2018 ▸). Finally, automated image segmentation and analysis enable to interrogate and to classify the 3D reconstructed volume.

Below, we first detail the instrumentation and optics of the cone- and parallel-beam configuration in Sections 2 and 3. Details on sample preparation and experimental workflow are given in Section 4. Section 5 presents the multiscale tomography results, before the paper closes with conclusions and outlook in Section 6.

## 2. GINIX – setups   

Experiments were carried out at the holotomography endstation GINIX, installed at the P10 beamline at PETRA III located at DESY (Salditt *et al.*, 2015 ▸). By tuning the 5 m undulator, the instrument can be operated in an X-ray energy range between 6 keV (first harmonic) and 15 keV (third harmonic). The beam is monochromated by either a channel-cut or a double-crystal monochromator [both Si(111)]. Here, two tomography configurations were combined to extend the range of length scales covered, one based on cone-beam the other on parallel-beam illumination. They were implemented in a side-by-side fashion, using the same tomographic stage and mounting. Experimental parameters used in this work are listed in Table 1 ▸ for both configurations. Here we used 13.8 keV in the cone-beam configuration, and 8 keV in the parallel-beam configuration for samples with a thickness (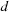 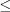 1 mm). In both cases, the channel-cut monochromator was used to achieve high pointing stability.

**Figure 1:**
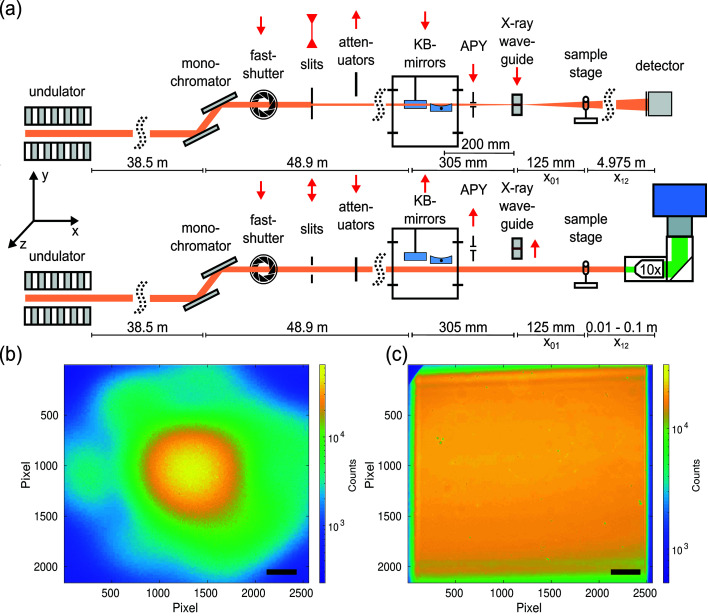
(*a*) Sketch of the X-ray beam path for cone (top) and parallel (bottom) beam configurations with the sample stage. (*b*) Flat-field of the cone-beam setup obtained with a germanium waveguide at 13.8 keV with 350 ms exposure time. (*c*) Flat-field of the parallel-beam setup at 13.8 keV [35 ms exposure time, 600 µm (24 times 25 µm) Si attenuator foils]. Exit window of the KB vacuum tank cut into the flat-field at the top left corner. Scale bars: (*b*) 1.95 mm in the detector plane corresponding to an angle of 0.38 mrad or 50 µm in the object plane; (*c*) 200 µm (detector and object plane).

Nanoscale tomography in cone-beam geometry, which is the standard use of the endstation, was carried out with a maximum FOV of 0.4 mm (h) × 0.35 mm (v) and an effective pixel size of 167 nm. The acquisition time for a single projection was 1 s. A single distance recording of 1500 projections including 100 empty beam images and 20 dark images took 42 min. The parallel beam configuration covered a 16 times larger FOV (1.6 mm × 1.4 mm) with an effective pixel size of 650 nm. The acquisition time for a single image was 35 ms. Both configurations and the corresponding empty beam images are shown in Fig. 1 ▸.

The configuration for the cone-beam geometry is detailed in Fig. 1 ▸(*a*, top): A pair of Kirkpatrick–Baez (KB) mirrors was used to focus the beam down to about 300 nm × 300 nm. To increase the spatial coherence and to filter the wavefront, an X-ray waveguide was placed in the focal plane of the KB mirrors. Between the KB mirrors and the waveguide, a 1 mm aperture in tungsten was used to block the remaining non-reflected primary beam as well as the single reflected beams. For 13.8 keV, a germanium waveguide was used with 200 µm optical depth and a channel diameter of 100 nm. Images were acquired with a Zyla HF 5.5 detector (Andor, USA) with a customized 15 µm-thick Gadox scintillator on a fiber optic plate coupled to a sCMOS chip with a 1:1 coupling and a pixel size of 6.5 µm. A detector point spread function of ∼2.0 pixels was determined at 10% of the detector modulation transfer function, measured at an inhouse setup. The detector was placed at a distance of 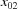 = 5100 mm behind the waveguide exit. Tomograms were acquired at several distances (Zabler *et al.*, 2005 ▸), starting with waveguide-to-sample distance 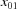 = 125 mm, corresponding to a magnification factor 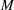 = 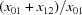 = 40.8, and an effective pixel size of 167 nm. A typical waveguide empty beam image is shown in Fig. 1 ▸(*b*). It exhibits a smooth far-field diffraction pattern with only a few contributing modes, and a particularly high photon flux of 5 × 10^9^ photon s^−1^.

The parallel-beam configuration is depicted in Fig. 1 ▸(*a*, bottom). Here, the high beam intensity in the sample plane enables fast tomographic scans with continuous motor movement. For this purpose, the sCMOS camera pco.edge 5.5 was used, which is able to acquire with maximum frame rate of 100 Hz for rolling shutter and fast scan mode. The camera was mounted on an Optique Peter system with a 50 µm-thick LuAG:Ce scintillator and a 10× magnifying microscope objective, resulting in an effective pixel size of 0.65 µm. Since the fastshutter (Cedrat technologies) movement was still too slow for the short acquisition time of 35 ms, it was removed together with the focusing elements, *i.e.* KB mirrors and waveguide, by moving these components out of the beam. To maximize the beam size for a larger FOV the upstream slits were opened to a size of 2 mm × 2 mm. To prevent scintillator damages due to the beam intensity, even if only minor changes in the sensitivity, the beam was attenuated by 100 µm (4 times 25 µm) single-crystal silicon attenuator foils for 8 keV and 600 µm (24 times 25 µm) silicon attenuator foils for 13.8 keV. An example flat-feld image with 35 ms exposure time for 13.8 keV is shown in Fig. 1(*c*) ▸.

When changing between both geometries, the tomographic stage was adjusted accordingly (lookup table), since the KB mirrors reflect the X-ray beam at an angle of 8 mrad in the *y*- and *z*-direction.

## 3. Data acquisition parallel beam setup   

**Figure 2:**
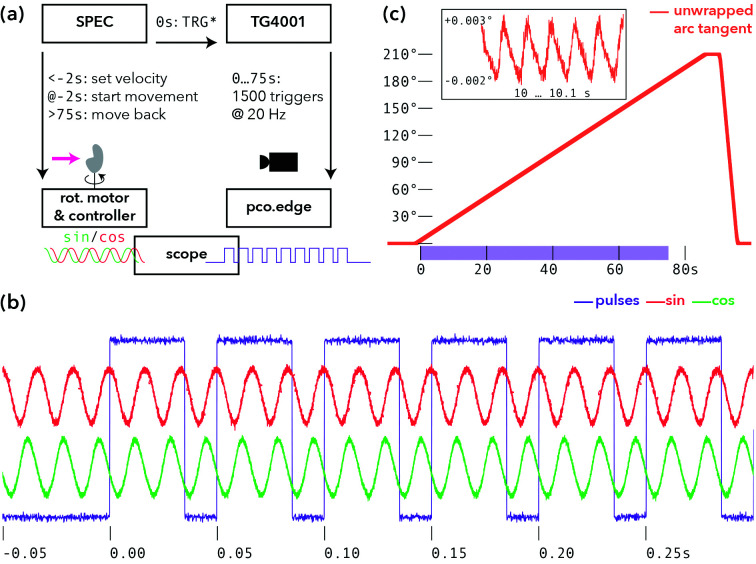
(*a*) Acquisition scheme for tomograms in the parallel-beam geometry (*SPEC*: control software; and TG4001: pulse generator). (*b*) Comparison of the rotary encoder signal (red/green) and the 35 ms trigger signals from the pulse generator (purple). Two projections are separated by 0.12°. Within 50 ms the rotary encoder returns three oscillations with a step size of 0.04° per oscillation. (*c*) Unwrapping the arctangent signal of the rotary encoder delivers a linear angular position curve over time.

Parallel-beam illumination with undulator radiation allows for short exposure times on the order of a few milliseconds. A step-wise rotation scan with acceleration, movement, deceleration, and settle times would result in an overhead of up to one order of magnitude in ‘wasted’ time. To adapt actual scanning speed to the true exposure time, a continuously rotating setup with burst-mode acquisitions [*i.e.* acquisitions to the solid-state drive (SSD) of the camera PC] by the detector was therefore implemented. In a first configuration, full tomograms consisting of 1500 projections within 180° were measured in less than two minutes (including setup time, residual motor overhead, and rotating back). In addition to the scan, empty beam and dark field acquisitions were recorded after the scan (500 flat images and 500 dark images). Although the pco.edge camera is capable of 100 Hz acquisition rate, we limited the rate to 20 Hz, corresponding to an exposure period of 35 ms and a read-out time of 15 ms. The camera was operated in external trigger mode. Tests showed that at this rate the read-out is reliable. The control and trigger system is sketched in Fig. 2 ▸(*a*).

Since the highly brilliant PETRA III parallel beam easily saturates the detector in less than 1 ms, attenuator foils needed to be deployed in order to achieve exposure times of 35 ms. Hence, significant further progress can be expected by detectors with internal frame-buffering and multi-kHz frame-rates. This can already routinely be reached by hybrid pixel detectors such as EIGER X 4M (Dectris, Baden-Daettwil, Switzerland) or Lambda (Pennicard *et al.*, 2012 ▸), but not by detectors with small pixel sizes, as required for parallel-beam tomography. For an accurate timing, the external trigger mode of the pco.edge was connected to an arbitrary function generator (TG4001 by Aim TTi, Huntingdon, UK), which provided 1500 TTL pulses of 35 ms duration at a rate of 50 ms, *i.e.* 20 Hz. Both incoming pulses and the frame clock of the detector were monitored by an oscilloscope (RTB 2004 by Rohde & Schwarz, Munich, Germany). Crucial for the success of a continuous tomography is a precise movement of the rotational axis. Within this work, the accurate position (*i.e.* absolute angle) is not of interest, but the spacing given by the angular velocity and frame rate has to be constant. Therefore, the analog encoder output signals of the Micos UPR 160-AIR (PI miCos, Eschbach, Germany) stage, which is controlled by a Micos Taurus unit, were fed into the oscilloscope as well. The UPR uses an incremental rotary encoder ‘RIK’ (Numerik Jena, Jena, Germany; miCos alias: ‘AE-051’), with 9000 steps per revolution, and delivers a 1/*V*_pp_sin/cos signal to the Taurus controller. One encoder step corresponds to a rotation by 0.04°; hence, one projection of the tomograms corresponds to three full encoder steps. Note that the sin/cos signal allows for an interpolation by a factor of 100; in fact, the measurement accuracy is stated to be 0.0004°, which coincides with the manufacturer specifications. Typical encoder signals including the trigger signal are shown in Fig. 2 ▸(*b*), here for a run of 1500 projections separated by 0.12° at 20 Hz. From the sin/cos encoder values, the current angular position can be obtained by taking the arctangent and unwrapping the data; Fig. 2 ▸(*c*) shows the values for one continuous tomogram; the shaded area highlights the actual measurement range.

Before conducting the tomographic scans, the angular movements of the UPR were measured for different velocities, and the linearity (constant rotational speed) was quantified. The data show that the acceleration phase was usually shorter than 1 s; with a safety margin of 100%, the trigger train to the pco.edge started 2 s after the movement of the rotation stage (UPR) was initiated. Note that the stage was commanded to perform a rotation of 210°, although the tomograms consist only of 180° within 30 s. In this way, the angular acceleration and deceleration phase was separated from the phase with linear rotation speed, where data were recorded. For the rotation back to 0°, the velocity was increased to about 210°/2.1 s. Comparison of encoder positions and nominal positions yielded a linearity of 2 × 10^−5^ (deviation of the slope when fitting measured versus nominal angle between). During rotation, the encoder values showed a sawtooth-like ripple of less than 0.006° at a frequency of about 60 Hz; see the inset in Fig. 2 ▸(*c*). Since this is not visible in the steady-state, we attributed this to the stage’s closed-loop operation; since the amplitude is smaller than 1/20 of the per-frame movement, it is of no concern for the data shown here.

## 4. Sample preparation and workflow   

**Figure 3:**
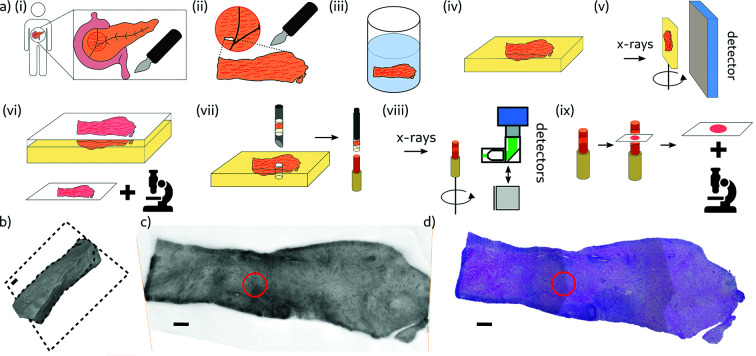
(*a*) Schematic of the sample preparation and the experimental workflow for the example of the PTA stained sample. (i) Surgery: removing human pancreatic cancer tissue. (ii) Sectioning: a tissue piece of a few millimetre size was sectioned from the removed tissue. (iii) Fixation and staining: tissue piece was fixated and stained in a phosphotungstic acid (PTA) staining solution. (iv) Paraffin embedding: a tissue piece was embedded in paraffin. (v) Inhouse µ-CT: tomograms of the tissue sample within the paraffin block were acquired at our laboratory phase-contrast µ-CT instrument with an effective pixel size of 5.3 µm. (vi) 2D histology: tissue slides of 2.5 µm thickness were cut from the top, stained by H&E (hematoxylin and eosin), and imaged with a microscope. (vii) Punch biopsy: 1 mm punch biopsy was taken from the sample by using a needle of 1 mm diameter, inserted into a polyimide tube of 1 mm diameter and mounted on a brass pin. (viii) Synchrotron CT: tomograms of the punch biopsy were taken at the synchrotron endstation GINIX. (ix) 2D histology of punch biopsy: after the tomographic scans, histology slides were taken from the punch biopsy for validation purposes. (*b*) 3D volume rendering of Sample B (PTA stained), Dashed lines mark the reconstructed slice shown in (*c*). (*c*) Virtual slice of the reconstructed 3D volume of an X-ray tomogram acquired at the laboratory setup. The red circle indicates the position of the punch biopsy. (*d*) Image of the histological slide of the sample, stained with H&E, corresponding to the virtual slice in (*c*). The red circle indicates the position of the punch biopsy. Scale bars: (*b*), (*c*) and (*d*) 600 µm.

The sample preparation and the workflow for the 3D virtual histology of two human PDAC tissues by X-ray phase-contrast tomography was divided into the following steps, illustrated in Fig. 3 ▸(*a*). (i) Surgery: the tumorous pancreatic tissue of two different patients was removed by pancreatic cancer surgeries. (ii) Sectioning: a small piece with a size of a few millimetres was sectioned from both removed tissues (Ethics statement: Nr. 5/10/17 UMG Goettingen). (iii) Fixation and staining: one sample (Sample A) was fixated in 4% Formalin without additional staining, while the other sample (Sample B) was fixated and stained in a phosphotungstic acid (PTA) staining solution as described before by Missbach-Guentner *et al.* (2018 ▸). Note that one of the technical goals in this study was to investigate how staining affects image quality and contrast and whether it is required in order to detect the morphological features. (iv) Paraffin embedding: the tissue samples were dehydrated in an ethanol series and then embedded in paraffin. (v) Inhouse µ-CT: tomograms of the tissue samples within the paraffin blocks were acquired at our home-built X-ray laboratory phase-contrast µ-CT instrument to obtain a 3D volume of the entire tissue pieces before further destructive preparation steps. The instrument was equipped with a liquid-metal anode (JXS D2, Excillum, Sweden), a tomographic sample tower (SmarAct, Germany) and a flat-panel detector (1512 CMOS Detector, PerkinElmer, USA) (Töpperwien *et al.*, 2017*b* ▸). Based on the geometrical magnification in a cone-beam geometry the effective pixel size was adjusted to 5.3 µm. The shape of the entire PTA stained sample (Sample B) is illustrated in Fig. 3 ▸(*b*) as a volume rendering. A virtual slice through the reconstructed volume indicated by the black dashed square is shown in Fig. 3 ▸(*c*). (vi) 2D histology: tissue slides of 2.5 µm thickness were cut from the top of the samples using a microtome, and then stained with H&E (hematoxylin and eosin). Notable morphological features of the tumor were defined. An image of the histology slice corresponding to the virtual slice from the inhouse µ-CT is shown in Fig. 3 ▸(*d*). Both images represented the tissue morphology with comparable quality. (vii) Punch biopsies: to fit into the detector FOV at the synchrotron setup and to decrease the X-ray absorption of the sample, 1 mm punch biopsies were taken from the samples by using a needle of 1 mm diameter (biopsy punch, Integra Miltex, Germany). The choice of the punch biopsies (red circle) was made based on the histology slice in Fig. 3 ▸(*d*), aiming for specific structures of pathologically relevance. The punch biopsies were inserted into a polyimide tube of 1 mm diameter and mounted on respective brass pins. (viii) Synchrotron CT: tomograms of both punch biopsies were recorded at the synchrotron endstation GINIX. Results and findings are discussed in the next section in detail. (ix) Validation by 2D histology: to confirm morphological structures visible in the synchrotron tomograms, tissue sections of 2.5 µm thickness were taken at controlled height positions of the punch biopsies, after recording the tomograms.

## 5. Results   

### 5.1. Phase-contrast formation and retrieval   

**Figure 4:**
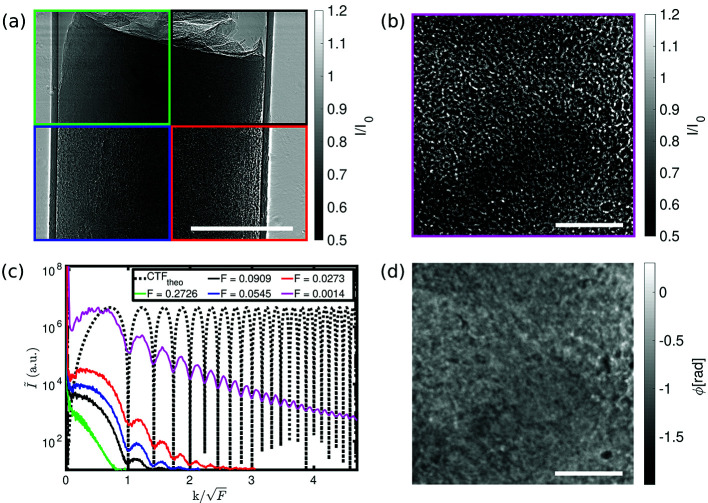
(*a*) Empty-beam corrected projections of an unstained human pancreatic tissue sample (Sample A) for four different propagations distances for the parallel-beam illumination in the direct-contact regime. Green: 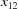 = 10 mm (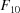 = 0.2726). Black: 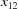 = 30 mm (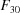 = 0.0909). Blue: 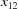 = 50 mm (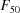 = 0.0545). Red: 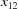 = 100 mm (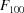 = 0.0273). Phase-contrast increases with decreasing Fresnel number. (*b*) Empty-beam corrected projection of the sample in (*a*) for the cone-beam illumination in the holographic regime. Magenta: 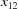 = 4975 mm (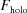 = 0.0014). (*c*) Power spectral densities of an squared area in the middle of the projected sample. The black dotted line shows the theoretical sinusoidal oscillations of the CTF. (*d*) CTF-reconstructed phase image of (*b*). Scale bars: (*a*) 500 µm and (*b*) and (*d*) 100 µm.

For propagation-based phase-contrast, interference effects and hence contrast is governed by the unitless Fresnel number 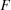 = 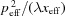, where λ denotes wavelength, 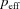 the effective pixel size, and 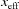 the effective propagation distance. The two setup configurations cover different regimes, as illustrated in Fig. 4 ▸. The parallel-beam configuration yields data in the so-called direct-contact regime (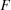 ≃ 1). Here, the phase-contrast is visible in the recorded images in the form of edge enhancement. This effect intensifies with increasing propagation distances, visualized in Fig. 4 ▸(*a*). The cone-beam geometry at high geometric magnification yields data in the holographic regime (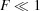), visible in the form of multiple overlaying interference fringes. An empty-beam corrected image is shown in Fig. 4 ▸(*b*). The power spectral densities (PSDs) for the five propagation distances (four parallel + one cone) are plotted in Fig. 4 ▸(*c*). Larger propagation distances led to more oscillations in the PSD. The shape is well described by the sin-weighted phase term of the contrast transfer function (CTF) (Cloetens *et al.*, 1999*b* ▸; Zabler *et al.*, 2005 ▸) with 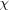 = 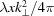,
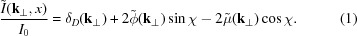
For this reason, phase retrieval was performed with the single-step CTF approach (Turner *et al.*, 2004 ▸),
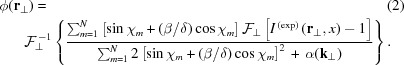
The reconstructed phase of the holographic projection Fig. 4(*b*) is shown in Fig. 4 ▸(*d*). Phase retrieval was performed for all projections based on *H olotomotoolbox* (Lohse *et al.*, 2020 ▸).

After the phase retrieval for the entire set of projections, the tomographic reconstruction was performed with the MATLAB implemented Iradon-function (Ram-Lak filter) for the parallel geometry and with the FDK-function of the *ASTRA* toolbox (Van Aarle *et al.*, 2015 ▸, 2016 ▸) for the cone-beam geometry. Tomograms were reconstructed from 1500 projections over 180° for both cone and parallel beam. Ring artifact correction according to the method of Ketcham (2006 ▸) was performed on every tomogram.

### 5.2. Virtual histology   

**Figure 5:**
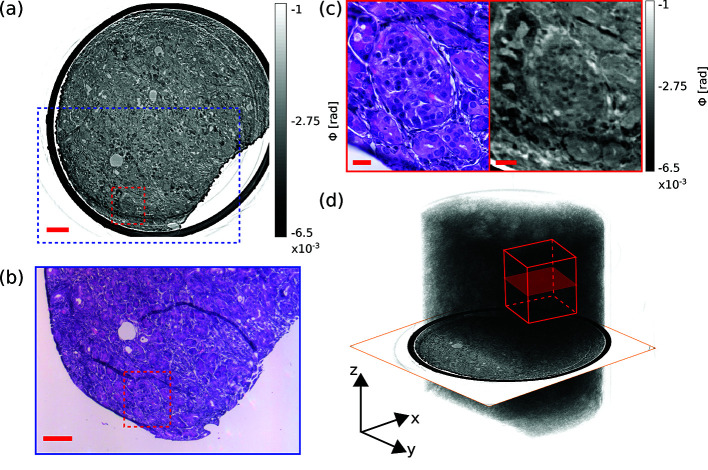
(*a*) A representative virtual histological slice in the *xy*-plane of Sample A obtained by the parallel-beam configuration with 10 mm propagation distance is shown. Gray values encode the reconstructed phase shifts. The dashed blue square indicates the area of the image in (*b*). The dashed red square indicates the area of the right-hand slice in (*c*). (*b*) For a correlative histological assessment by H&E staining, a slice was taken and stained after the tomographic acquisition to identify structures of the virtual slice. The dashed red square indicates the left-hand slice in (*c*). (*c*) Comparison of morphological structures in the magnified areas of (*a*) and (*b*) indicating that the morphology is matched well. With the H&E stained section, the cluster of cells in the center of the ROI was confirmed to be an islet of Langerhans (left). The same islet of Langerhans is also visible in the virtual slice (right). (*d*) 3D volume rendering of the entire Sample A. Inserted are both the virtual slice of (*a*) and an exemplary volume for the high-resolution tomogram. Scale bars in (*a*) and (*b*): 100 µm; and (*c*) 20 µm.

Two samples of human pancreatic cancer tissue, originating from different patients, were investigated. Sample A represents an unstained 1 mm punch biopsy taken from putative healthy tissue. Heavy metal staining often increases the image contrast in conventional X-ray imaging based on absorption. To check the image contrast of a stained sample in the context of the present phase-contrast tomography, Sample B was a PTA stained 1 mm punch biopsy, consisting mainly of tumor tissue. Four different tomograms were acquired, including one tomogram in the parallel-beam configuration and one in the cone-beam configuration for both samples, respectively. The image quality was evaluated and morphological features were assessed, and compared with conventional 2D histology, illustrated in Fig. 5 ▸. An example slice in the *xy*-plane for virtual histology of Sample A, obtained using the parallel-beam configuration with 10 mm propagation distance, is shown in Fig. 5 ▸(*a*). The pancreatic tumor tissue is surrounded by the polyimide tube (black) and did not fill the entire tube, since there was an air-filled gap between tissue and tube in the bottom right. Contrast of the reconstruction was sufficient to investigate the morphology of the tissue. Here, we focused on a cluster of specific cells within the normal pancreatic tissue, the islet of Langerhans, which is a distinct shaped accumulation of endocrine cells, located within the exocrine pancreatic parenchyma, responsible for the endocrine function of the pancreas. The most common islet cell, the beta cell, produces insulin, the primary hormone regulating glucose metabolism. A representative islet of Langerhans is located in the red marked area in Fig. 5 ▸(*a*). Figure 5 ▸(*b*) shows approximately the same slice (adjusted by hand), presented as an image of an H&E stained section obtained by routine histology after the tomographic acquisition. The image corresponds to the blue marked area in Fig. 5 ▸(*a*). Comparative inspection, as illustrated in Fig. 5 ▸(*c*), revealed that both slices show the same morphology. Slight differences of shapes of the structures, *i.e.* the bright duct at the middle of the right side, and of the image contrast, *i.e.* the dark surrounding at the top left of the X-ray image, were visible. Minor shape differences can be explained by the fact that both slices are only approximately identical, as well as by deformations induced during sectioning and staining. Contrast differences could result from the structure specific H&E staining in the histological image compared with the unstained sample in the X-ray image. The H&E staining confirmed that the cluster of cells in the central [Fig. 5 ▸(*a*), marked red area] is an islet of Langerhans. Figure 5 ▸(*d*) visualizes the entire 3D dataset of Sample A as a volume rendering. The dataset covers a volume of 3.888 mm^3^ in total. The virtual slice of Fig. 5 ▸(*a*) is included, and the volume of the high-resolution scan is indicated by the red cube with a volume of 0.035 mm^3^. Note, however, that the drawing is intended to illustrate a comparison of reconstruction volume size – the positions of the high-resolution slices shown in the following below may not coincide. The cylindrical shape of the punch biopsy is clearly visible. The polyimide tube was masked out for the purpose of visualization. It was difficult to distinguish specific structures in the 3D rendered volume, due the homogeneous morphological structure of the sample. The paraffin-filled areas stand out from the surrounding as brighter blobs in this large-scale rendering, visible for example in the top region.

**Figure 6:**
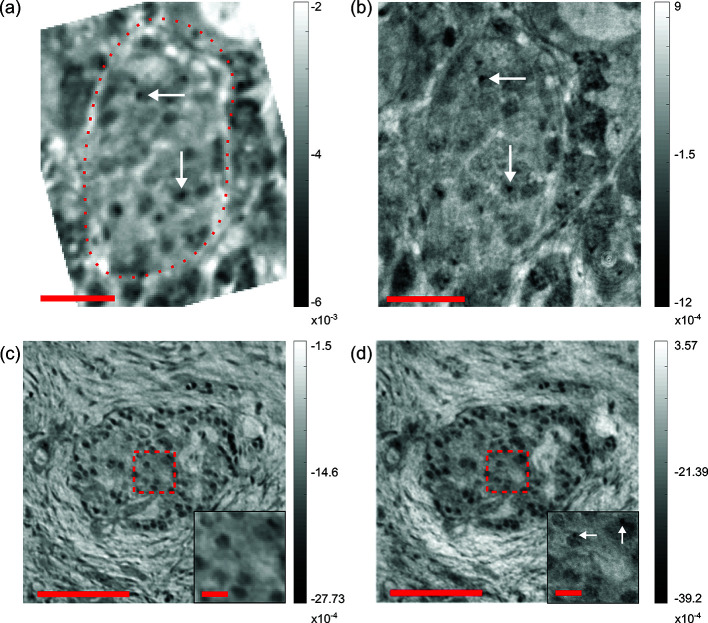
(*a*) ROI centered around an islet of Langerhans, identified within reconstructed *xy*-slices of the unstained pancreatic cancer tissue punch biopsy (Sample A), from parallel-beam scan. The dashed red line indicates the islet of Langerhans. (*b*) ROI within reconstructed *xy*-slice of Sample A, from cone-beam scan. Resolution is enhanced and finer structures are resolved. Cell nuclei are marked by white arrows. (*c*) ROI within a reconstructed *xy*-slice of the PTA-stained PDAC tissue of biopsy Sample B, from parallel-beam scan. Magnified area: single cells located inside the islet of Langerhans are resolved. (*d*) ROI within a reconstructed *xy*-slices of Sample B, from cone-beam scan. Single-cell nuclei are visible. Magnified area: nuclei of cells which form the islet of Langerhans are resolved, marked by the white arrows. Scale bars: (*a*, *b*) 25 µm, (*c*, *d*) 75 µm and magnified areas 10 µm.

Next, the image quality of parallel- and cone-beam setups was assessed by comparing the reconstructed slices of the same area of an islet of Langerhans for Sample A and Sample B, respectively, as illustrated in Fig. 6 ▸. A reconstructed slice of Sample A in the *xy*-plane obtained from the parallel beam geometry is shown in Fig. 6 ▸(*a*). The image contrast is sufficient to identify the islet of Langerhans, which is marked by the dashed red line. The image of the same islet of Langerhans, obtained in the cone-beam geometry, is shown in Fig. 6 ▸(*b*). As expected, the resolution is higher (pixel size 167 nm) compared with the parallel beam reconstruction (pixel size 650 nm). Finer structures such as small collagen fibers from surrounding connective tissue at the top edge of the islet of Langerhans are visible in the cone-beam reconstruction. In both images, the contrast is high enough to locate nuclei in some cells.

In order to increase the image contrast, we also recorded tomograms of the PTA stained punch biopsy (Sample B). The parallel-beam reconstruction is shown in Fig. 6 ▸(*c*). The entire islet of Langerhans as well as single-cell bodies were visualized with higher contrast compared with the unstained Sample A. Figure 6 ▸(*d*) shows the same slice (approximately, adjusted by hand) as Fig. 6 ▸(*c*), obtained with the cone-beam configuration. The difference in the resolution is highlighted by the insets. The resolution of the cone-beam reconstruction [Fig. 6 ▸(*d*)] was sufficient to resolve single-cell nuclei, which were not visible in the parallel-beam reconstruction.

To quantify the resolution, the Fourier shell correlation (FSC) (Van Heel & Schatz, 2005 ▸) was computed for the parallel-beam volumes, indicating a half period resolution of 0.90 µm for Sample A and 0.82 µm for Sample B.

### 5.3. 3D properties of an islet of Langerhans   

**Figure 7:**
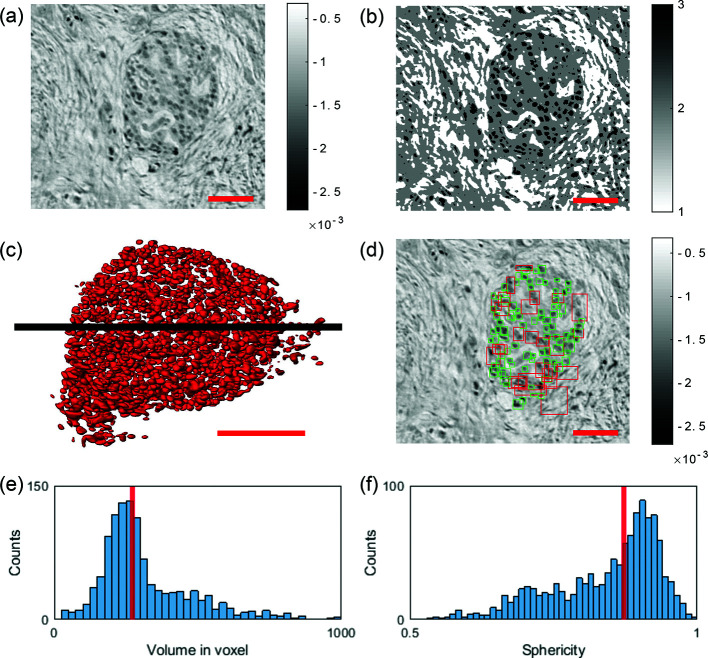
(*a*) Reconstructed slice in the *xy* plane of the PTA-stained punch biopsy (Sample B) obtained by the parallel-beam illumination. (*b*) Pixel segmentation into three classes via *ilastik*. (*c*) 3D rendering of the segmented cells located within the islet of Langerhans by their manual selection. (*d*) Boundary boxes of the segmented cells. Each square marks one component. If the width of one component is larger than 20 pixels in one dimension, it is declared as false (red squares). Squares of components with the correct size are marked green. (*e*) Cell volume distribution of the selected cells [green marked in (*d*)]. Note that the median volume size of a single cell is 271 voxel. (*f*) Distribution of the cell sphericity Ψ of the selected cells [green marked in (*d*)]. Note that the median cell sphericity is 0.87. Scale bars in (*a*), (*b*), (*c*) and (*d*): 50 µm.

Next, we used the 3D dataset of Sample B, obtained in parallel-beam geometry, to analyze the shape of the islet of Langerhans in more detail, shown in Fig. 7 ▸. Since the resolution of the parallel-beam data was sufficient for segmentation of cells, and higher resolution with sub-cellular features can even be a source of complication for cell segmentation, we chose the parallel-beam dataset for this task.

The segmentation was performed using the software *ilastik* (Berg *et al.*, 2019 ▸). Cells located inside the islet, shown in the reconstructed slice in Fig. 7 ▸(*a*), were segmented by interactively marking a few cells, followed by an automatic completion of the entire volume based on machine-learning algorithms. Figure 7 ▸(*b*) shows the same *xy*-slice of the segmented volume. Segmentation comprises three tissue classes: 1 = high gray-value stroma, 2 = low gray-value stroma, 3 = cells. The segmentation step was completed by a masking step, to exclude false positive cells outside the islet. Figure 7 ▸(*c*) shows a 3D rendering of all segmented cells located inside the islet.

The segmented volume was then further analyzed with Matlab. Cells were identified using the Matlab function bwconncomp(), which detects connected components in a binary image/volume. With an input parameter ‘Connectivity’ of 6, which takes the 6 nearest voxel into account, 1309 single cells were counted inside the islet of Langerhans without any corrections. Visual inspection shows that some segmentation components include more than one cell, due to a connection in the segmentation. To filter the connected cells out for the subsequent histograms, we introduced a volume threshold. Based on an estimated maximum cell size of 20 pixels (= 13 µm), all cells with a larger size in one of the *x*-, *y*-, *z*-directions were subjected to a veto, as illustrated in Fig. 7 ▸(*d*). The squares represent the boundary boxes of each segmented component in 3D. Green squares contain connected binary components below 20 pixel width and red squares comprise larger connected components within the volume. In this way, 139 objects were found to be too large. In order to count the connected cells inside the larger objects (consisting of seemingly connected cells), we divided the corresponding volume of all segmented objects above threshold (224865 voxel) by the median volume of a single cell (271 voxel), obtained from the distribution of properly segmented cells (below threshold), as shown in Fig. 7 ▸(*e*). This led in this example to a total number of 2000 cells located within the islet of Langerhans, which is in good agreement with the literature (Welsch & Kummer, 2018 ▸).

Volume, surface area and boundary boxes of each component were determined by using the Matlab function regionprops3(). As a further morphological measure, the sphericity of each cell was computed and calculated by the following definition (Wadell, 1932 ▸),
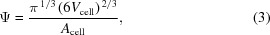
with the cell volume 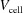 and the cell surface area 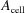.

The sphericity distribution is shown in Fig. 7 ▸(*f*).

### 5.4. Anisotropy parameter   

Since the tumorous pancreatic tissue is characterized by an abundance of collagen fibers known as typical desmoplastic reaction (intratumoral fibrosis), the 3D tissue structure shows a pronounced fiber orientation. Based on this one specific property of the tumorous tissue, our goal was to introduce a parameter, quantifying the tumor-induced fibrotic morphology, *i.e.* the degree to which the tissue is affected.

To this end, the anisotropy parameter Ω was used, defined as a scalar field. Ω describes the local degree of orientation for a chosen subvolume 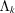 centered around the voxel in question. Its value is based on the three eigenvalues 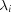 of the structure tensor ST as in Reichardt *et al.* (2019 ▸). By shifting the subvolumes voxelwise, every voxel of the tomographic volume can be allocated with an Ω-value. The structure tensor can be defined as the sum (or average) over the subvolume 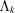 of the multiplication of the intensity gradient components of each voxel,
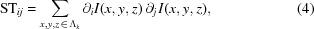
from which the Eigenvalues 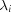 are calculated by
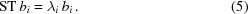
The anisotropy parameter Ω can then be defined as
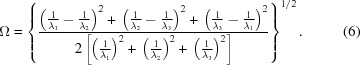
Ω evaluates to 0 for an isotropic structure (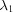 ≃ 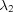 ≃ 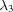), while for a highly anisotropic structure (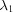 ≃ 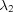 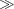 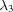) Ω converges to 1. For the following results 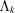 was chosen with a subvolume size of 40 pixel^3^. Subvolume 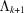 was shifted by 10 pixel with respect to 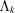, leading to a binning of the volume by a factor of ten.

 The concept of the Ω measure was demonstrated on three volumes with different collagen abundance:

Volume (1) – containing mostly healthy tissue, consisting of glandular tissue (blue);

Volume (2) – containing in part intratumoral fibrosis tissue (yellow);

Volume (3) – containing large areas of intratumoral fibrosis tissue (red).

**Figure 8:**
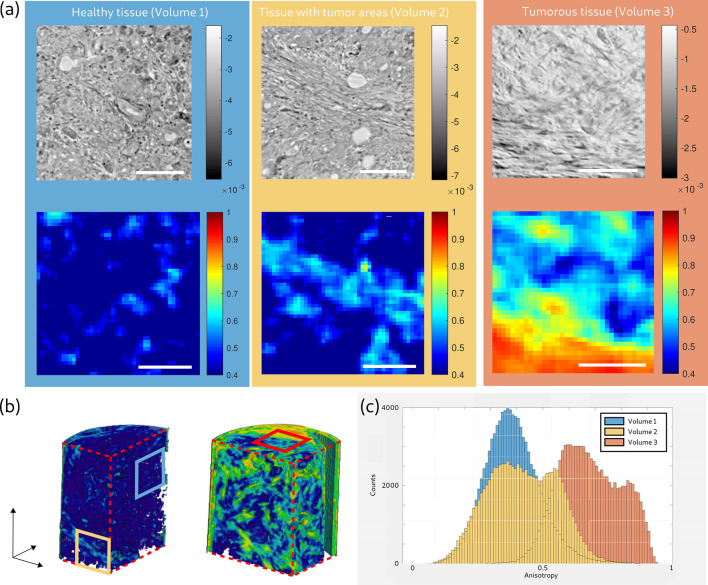
Results of the anisotropy parameter Ω for three example volumes of pancreatic tumor tissue. (*a*) Virtual slices (top) obtained from the parallel-beam configuration and corresponding anisotropy maps (bottom) for Volume (1), containing mostly healthy, glandular tissue (blue); Volume (2), containing partially tumorous tissue (yellow); and Volume (3), containing mostly tumorous tissue with intratumoral fibrosis (red). Note that the blue and yellow marked slices were chosen from the 3D reconstruction of Sample A, while the red marked slice was chosen from the 3D reconstruction of Sample B. (*b*) 3D visualization of the anisotropy parameter Ω of Sample A (left) and Sample B (right). Blue, yellow and red frames mark the positions of the corresponding slices in (*a*). (*c*) Histograms of the three anisotropy volumes. Ω values of the histograms increase with the presence of collagen fibers inside the volume. Scale bars: 100 µm.

Due to the morphology of both samples, Volumes (1) and (2) were selected from the parallel-beam reconstruction of Sample A, while Volume (3) was selected from the parallel-beam reconstruction of Sample B. Figure 8 ▸(*a*) shows the virtual slice from the parallel beam reconstruction (top), and the corresponding anisotropy map (bottom). The position within the biopsy for the volumes is shown in Fig. 8 ▸(*b*), indicated by the colored frames. Visual inspection of the virtual slice and the corresponding anisotropy map demonstrate how the fibrous structures of the tumor are represented by an increased anisotropy parameter Ω.

The three volumes are compared by their Ω-histograms in Fig. 8 ▸(*c*). The histogram of the healthy tissue Volume (1) is Gaussian distributed with a mean value of 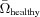 = 0.38 and a standard deviation of 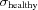 = 0.104. Volume (2) still contains healthy tissue, but the presence of collagen fibers increases as shown in the virtual slice, and the distribution of the histogram is broadened and shifted towards higher Ω-values (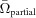 = 0.43 and 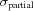 = 0.132). In the histogram of Volume (3), which contained mostly tumorous tissue, the Ω-values of the histogram are further shifted to higher (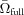 = 0.68 and 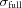 = 0.118). These three examples indicate that the tissue volumes can be classified based on their Ω histograms into different stages of affected tissue. The Ω-values increased with the increased presence of fiber, as expected, and the average value 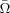 of a biopsy might therefore serve as a suitable biomarker to classify the tumor. To this end, future work should extend this analysis to a representative number of cases, and a validation based on the correlative imaging technique.

## 6. Conclusion and outlook   

In this work we have performed multiscale 3D virtual histology of human pancreatic biopsies combining two configurations of X-ray propagation imaging with synchrotron radiation. Since cancer surgery is often confronted with removal of substantial tissue volumes, 3D patho-histology automatically faces a multiscale challenge.

Here we have met this challenge by first scanning a large tissue block by inhouse µ-CT to obtain a large-scale overview. In addition to the morphological assessment by µ-CT, the paraffin-embedded tissue block was cut by a microtome, H&E stained, and morphologically notable regions of the tumor were defined. This pre-characterization allowed a precise selection of tissue regions, which were then targeted by punch biopsies, *i.e.* cylindrical volumes of 1 mm diameter were selected for subsequent high-resolution analysis at the synchrotron endstation GINIX. Here, the punch biopsies were first scanned by parallel-beam tomography, followed by further zooms into regions-of-interest by cone-beam holotomography.

Approaches of 3D multiscale phase-contrast X-ray tomography with synchrotron radiation have been realized before, *e.g.* by Dejea *et al.* (2019 ▸) at the TOMCAT beamline with an effective pixel size of 5.8 µm for low and 0.65 µm for high-resolution tomograms, respectively. Massimi *et al.* (2019 ▸) used two different beamline endstations for scans with a pixel size of 1.62 µm at TOMCAT and 50 nm at ID16A-ESRF. Contrarily, here both modalities were integrated side-by-side and hence available in close spatial and temporal proximity during a single beam time.

As a proof-of-concept of this multiscale 3D virtual histology approach, we chose pancreatic tumor samples obtained from pancreas cancer surgery, a tumor type exhibiting morphological alterations with very heterogeneous features. As a result, we could show that the degree of intratumoral fibrosis could be quantified in regions containing pancreatic tumors consisting of tumor cells and areas of desmoplastic reaction. To this end, we used an order parameter for tissue anisotropy, designed to measure the fibrous structure based on the structure tensor. As a further result, we noted that the islets of Langerhans, a cluster of endocrine cells located within pancreatic tissue, are clearly detected in tomograms, even in unstained paraffin-embedded tissue and the parallel-beam configuration. In the case of PTA staining, the contrast of the islet is increased. Much finer structures, *e.g.* cell nuclei, can be resolved in the zoom tomogram of the islets by holotomography. Comparison with classical histology on selected slices, sectioned after the synchrotron beam time, confirmed the identification of the islets. Importantly, tomography allows for enhanced quantification of the 3D structure. For the present example of a representative islet of Langerhans, 2000 endocrine cells were counted by semi-automatic segmentation with a median volume size of 74 µm^3^ and a median cell sphericity of 0.87. In future, similar analysis might be very helpful to obtain more insights into the histopathology of type 1 diabetes by analysing the islets of Langerhans in 3D within the pancreas tissue of these patients. Furthermore, specific features identified by tomography in large tissue volumes can be subsequently studied by well controlled histological sections, taking advantage of the possibility to characterize the morphological structures of interest by further immunohistochemical analysis. Note that coverage of comparably large volumes with micrometre- or sub-micrometre resolution is nearly impossible by parallel sections, or at least extremely tedious.

Since previous virtual histology applications at synchrotron as well as at laboratory setups were largely limited to small-animal models (Albers *et al.*, 2018 ▸; Busse *et al.*, 2018 ▸; Dejea *et al.*, 2019 ▸) or to *post mortem* human tissue (Töpperwien *et al.*, 2018 ▸; Buscema *et al.*, 2019 ▸; Vågberg *et al.*, 2018 ▸; Garcia-Canadilla *et al.*, 2018 ▸), the extension of the imaging technique to the 3D assessment of clinical biopsies (Norvik *et al.*, 2020 ▸; Katsamenis *et al.*, 2019 ▸) is now particular timely. Beyond research of tumor patho-physiology, the technique may even be applied in future in a clinical context with potential impact on the diagnosis of and on clinical treatment decisions. By identifying novel patho-histological features in 3D, tumors might be characterized in more detail, enabling to classify tumors more precisely in subgroups to allocate patients to the best treatment option. The novel information from phase-contrast X-ray tomography includes volumetric and shape measurements, not accessible by classical patho-histology. Since 3D virtual histology offers the advantage of large volume throughput, high resolution, as well as compatibility with systematic and automated analysis, we believe that this virtual histology could be integrated into biomedical workflows.

In contrast to standard 2D imaging of selected histological sections, which sample the tissue structures only in an exemplary manner, the tissue can be probed in its full dimensionality and without gaps. At the same time, this calls for a multiscale high-throughput approach, which we have demonstrated here at the GINIX endstation of the coherence beamline P10/PETRA III. A parallel-beam configuration for overview tomograms has been implemented with high volume throughput on the order of 0.01 mm^3^ s^−1^ and sufficient image quality for single cell segmentation. This was then complemented by cone-beam configuration with high geometrical magnification for high-resolution scans of selected regions-of-interest (42 min for a single-distance tomogram).

For the immediate future, straightforward technical improvements can be envisioned. With novel waveguide design, exposure times of 350 ms per projection are possible, which makes it reasonable to also implement continuous rotation for the tomography scans in cone-beam geometry. A reduction in scan time by a factor of 3–5 is realistic. Further, different staining and embedding protocols can be implemented and validated to find the best compromise between image contrast and structure alterations by the sample preparation. Moreover, characteristic morphological structures of the normal and tumorous pancreatic tissue can be analyzed in more detail and segmented including blood vessels or intact pancreatic acini and glandular ducts as well as areas of tumor cells and of immunological response.

Beyond these obvious extensions, more important steps are studies with a high number of tumorous samples to obtain convincing parameters and thresholds for the structural metrics with respect to tumor classification. Given successful foundation work and clinical trials as an important next step, 3D virtual histology of cancer biopsies could become effective for more precise diagnosis followed by the optimal selection of treatment in two possible scenarios. In the first scenario, the synchrotron work would be translated to laboratory µ-CT (see the supporting information for a comparison of present image quality), for which it could provide ground truth calibration (for selected samples when needed), while the compact instruments would be used in a clinical environment, possibly even in the form of fast tomography to characterize tissues during surgical procedures. In a second scenario, fully dedicated endstations with automated measurement, evaluation and reporting would operate with biopsy express delivery, so that results can be presented to the clinicians within a time frame of 2–3 days, similar to other laboratory tests. Both scenarios will require further demonstrations of possible insights and diagnostic benefits. The technical and instrumental requirements for this research have now been provided.

## 7. Related literature   

The following references, not cited in the main body of the paper, have been cited in the supporting information: De Witte *et al.* (2009) ▸; Reichardt *et al.* (2017) ▸.

## Supplementary Material

Supporting Information showing a comparison of synchrotron parallel beam reconstruction and inhouse microCT. DOI: 10.1107/S1600577520011327/tv5015sup1.pdf

## Figures and Tables

**Table 1:** Experimental setup parameters

	Cone geometry	Parallel geometry
FOV	0.4 mm × 0.35 mm	1.6 mm × 1.4 mm
Pixel size	167 nm	650 nm
*x*_01_	125 mm	–
*x*_12_	4975 mm	10–100 mm
Regime	Holographic	Direct contrast
Rotation	Start–stop	Continuous
Exposure	1 s	0.035 s
Total exposure	42 min	75 s
Volumetric flow rate	1.93 × 10^4^ µm^3^ s^−1^	3.75 × 10^7^ µm^3^ s^−1^
