# Supplementary material for: 3D virtual histology of human pancreatic tissue by multiscale phase-contrast X-ray tomography
Source: J Synchrotron Radiat. 2020 Oct 23;27(Pt 6):1707–19. doi: 10.1107/S1600577520011327 (PMC7642968; doi:10.1107/S1600577520011327)
Supplement: Supplementary file 1 [file s-27-01707-sup1.pdf]

3d virtual histology of human pancreatic tissue by multiscale phase-contrast x-ray tomography: Supplemental Information

Jasper Frohn,[a] Diana Pinkert-Leetsch,[b,c] Jeannine Missbach-Güntner,[b] Marius Reichardt,[a] Markus Osterhoff,[a] Frauke Alves[b,c,d] and Tim Salditt [a,e]*

[a] *Institute for X-ray physics, University Göttingen, Friedrich-Hund-Platz 1, Göttingen, 37077, Germany,* [b] *Institute of Diagnostic and Interventional Radiology, University Medical Center Göttingen, Robert Koch Str. 40, Göttingen, 37075, Germany,* [c] *Max Planck Institute for Experimental Medicine, Group of Translational Molecular Imaging, Hermann-Rein-Straße 3, 37075 Göttingen, Germany,* [d] *Clinic of Hematology and Medical Oncology, University Medical Center Göttingen, Robert Koch Str. 40, 37075, Göttingen, Germany, and* [e] *Multiscale Bioimaging: From Molecular Machines to Networks of Excitable Cells (MBExC).*

E-mail: tsaldit@gwdg.de

In this supplemental material, we briefly present the performance of a table-top inhouse phase-contrast x-ray tomography system, in view of a possible future translation from synchrotron facilities to compact inhouse μCT setups. As described in the outlook of the main article, future work could be directed at integration of phase-contrast x-ray tomography into clinical practice (pathology of biopsies from tumor surgery) as a supporting imaging modality. Presently achievable image quality at custom built μCT setups, as illustrated in the comparison of Fig. 1 proves that 3D

information of the tissue morphology can already be gained, albeit at much longer scantimes and lower resolution compared to the synchrotron results. In future, synchrotron results could provide a ground truth verification for inhouse developments.

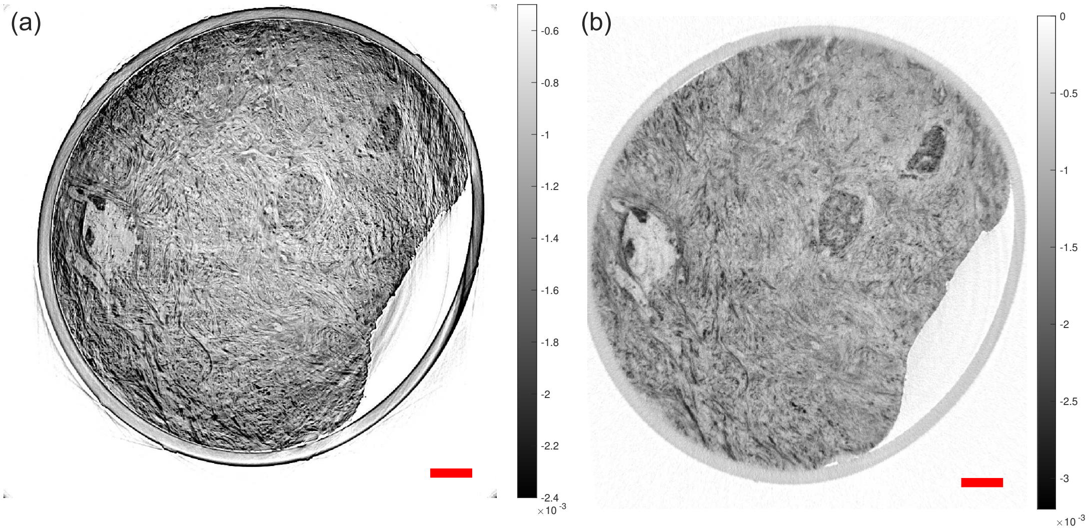

Fig. 1. Inhouse phase-contrast x-ray tomography.Comparison of virtual slices of **Sample B**. (a) Virtual slice obtained by the parallel beam configuration at the synchrotron setup. (b) Virtual slice obtained by the inhouse setup. Scale bars are 100 µm.

Inhouse setup: The home-built phase-contrast micro-CT setup is described in detail in (Reichardt et al., 2017). It consists of a rotating copper anode (Rigaku mm007) with an acceleration voltage of 40 kV and a tube current of 30 mA. The sample is positioned 500mm behind the focal spot. The circular spot size is 70 mm. Projection images are recorded with a high resolution (pixel size 550 nm) scintillator-based x-ray camera (XSight Micron, Rigaku), placed 5mm behind the sample. In this arrangement blur by the source spot is minimized, while spatial coherence and propagation distance is sufficient to observe edge enhancement. The projections were binned by a factor of 2 leading to a pixel size of 1.1 µm. Phase retrieval was performed by the BAC-algorithm (De Witte et al., 2009).

Experimental parameters: The tomographic acquistion consists of 1001 projec-

tions (over 180°) + 25 flat images + 10 dark images with an exposure time of 1 minute per image. This results in a total acquisition time of $\sim$ 18 h.

Results: Virtual histology of **Sample B** is illustrated in Fig. 1 for the parallel beam configuration in (a) and the inhouse setup in (b). The general shape of the sample is clearly comparable and large features such as the two islets of Langerhans are identifiable. With a pixel size of 1.1 µm of the inhouse setup, the resolution is clearly lower compared to the parallel beam synchrotron results, and the noise level is much higher.

References

De Witte, Y., Boone, M., Vlassenbroeck, J., Dierick, M. & Van Hoorebeke, L. (2009). JOSA A, **26**(4), 890–894.

Reichardt, M., Frohn, J., Töpperwien, M., Nicolas, J.-D., Markus, A., Alves, F. & Salditt, T. (2017). In Developments in X-Ray Tomography XI, vol. 10391, p. 1039105. International Society for Optics and Photonics.
